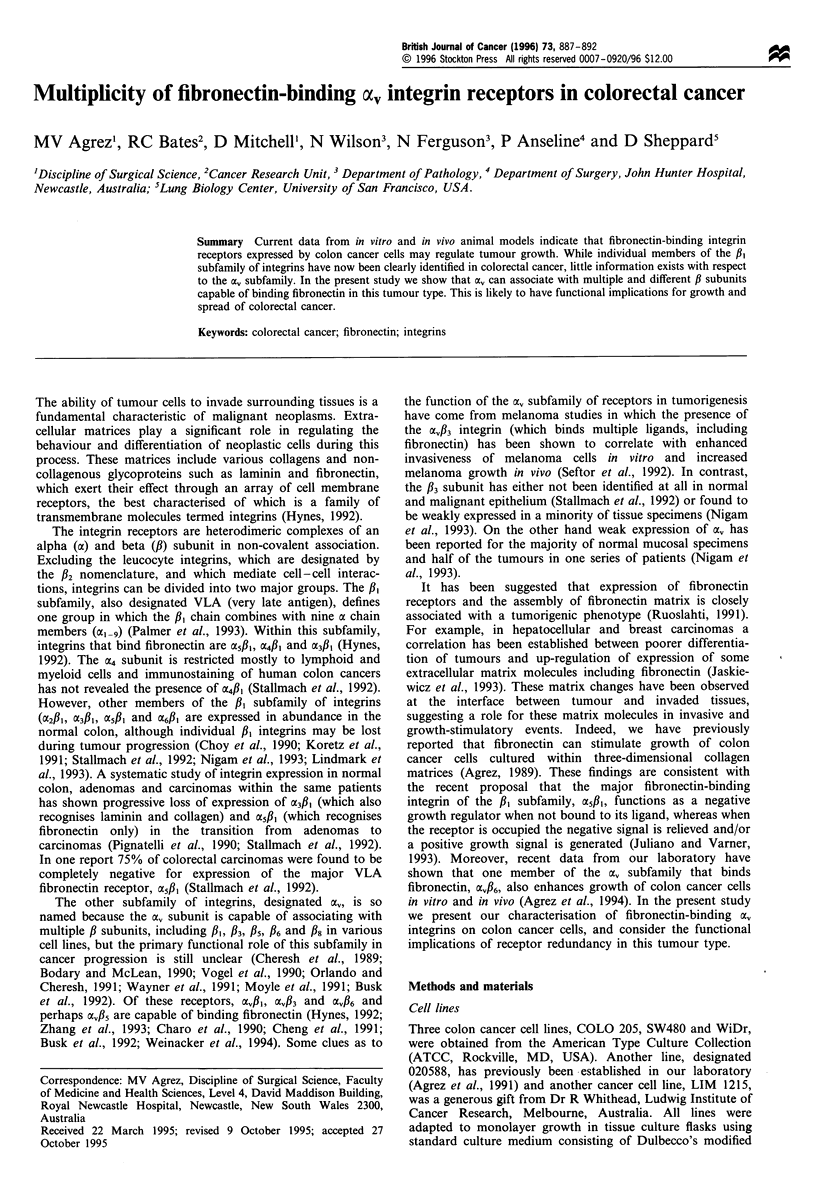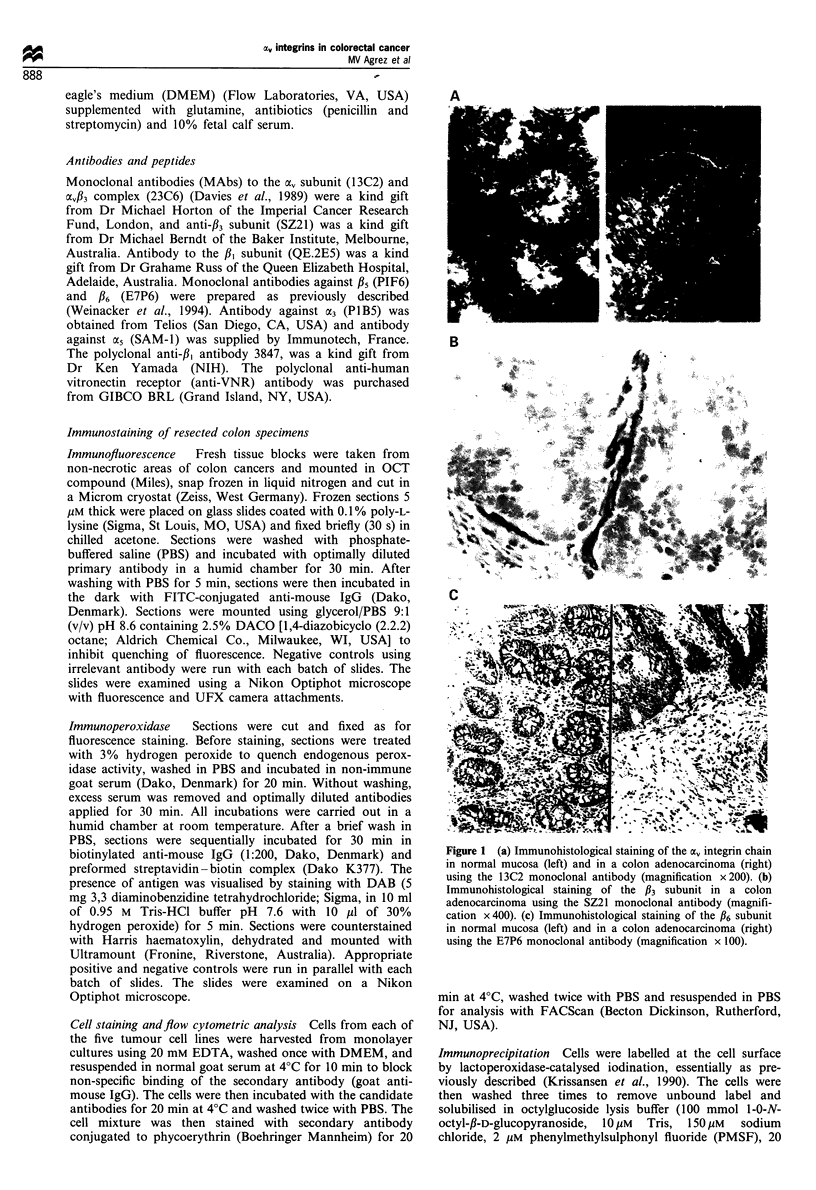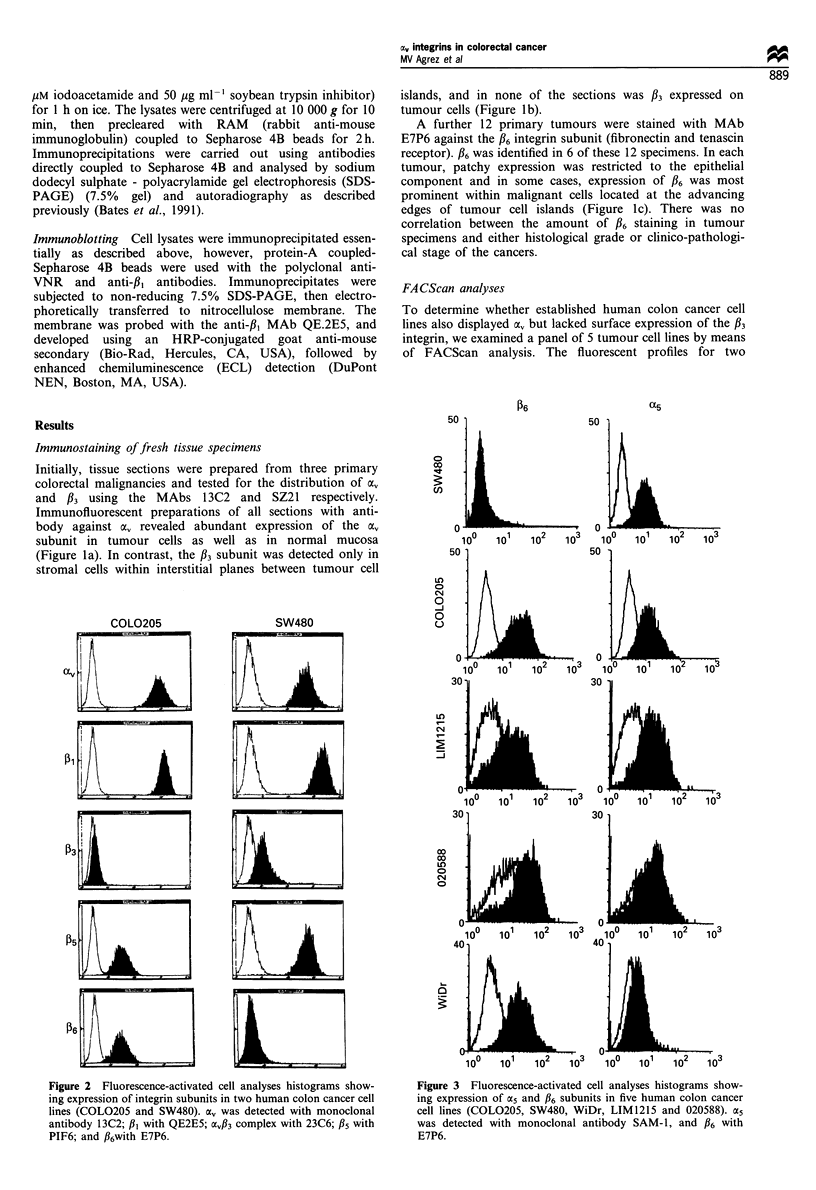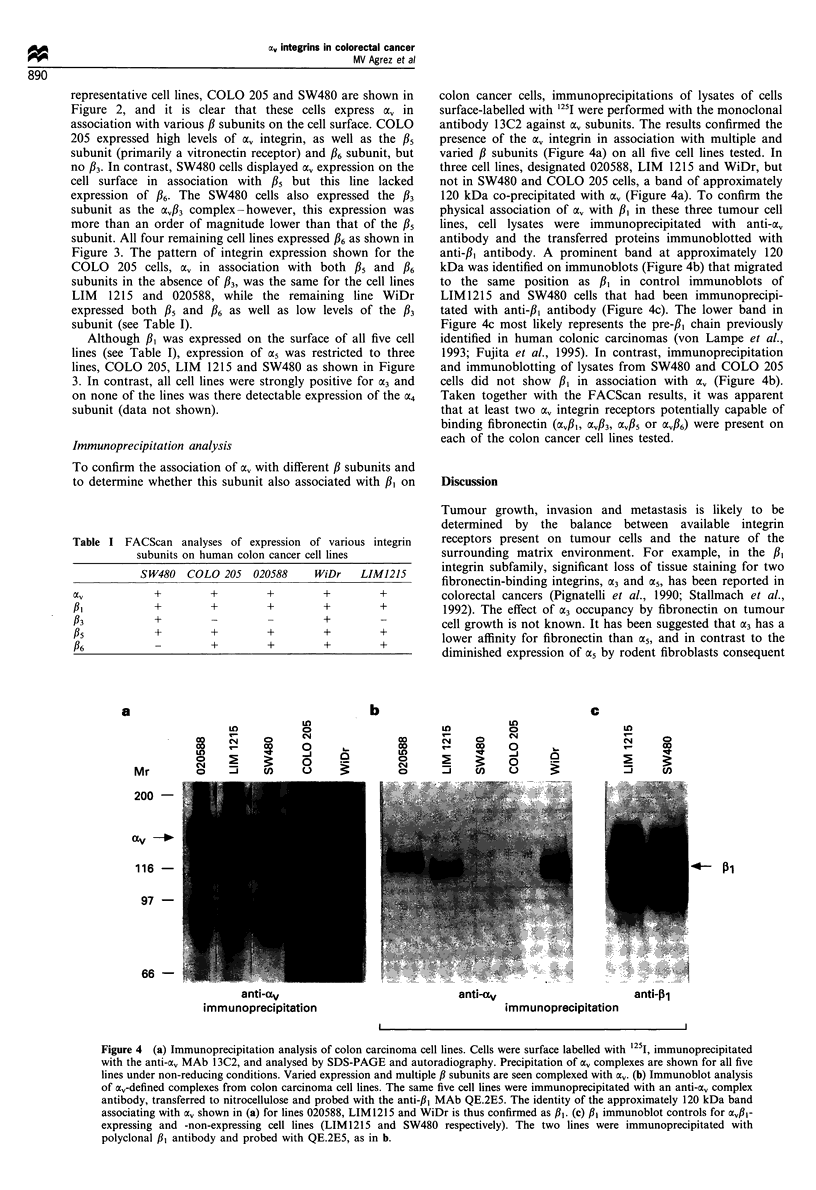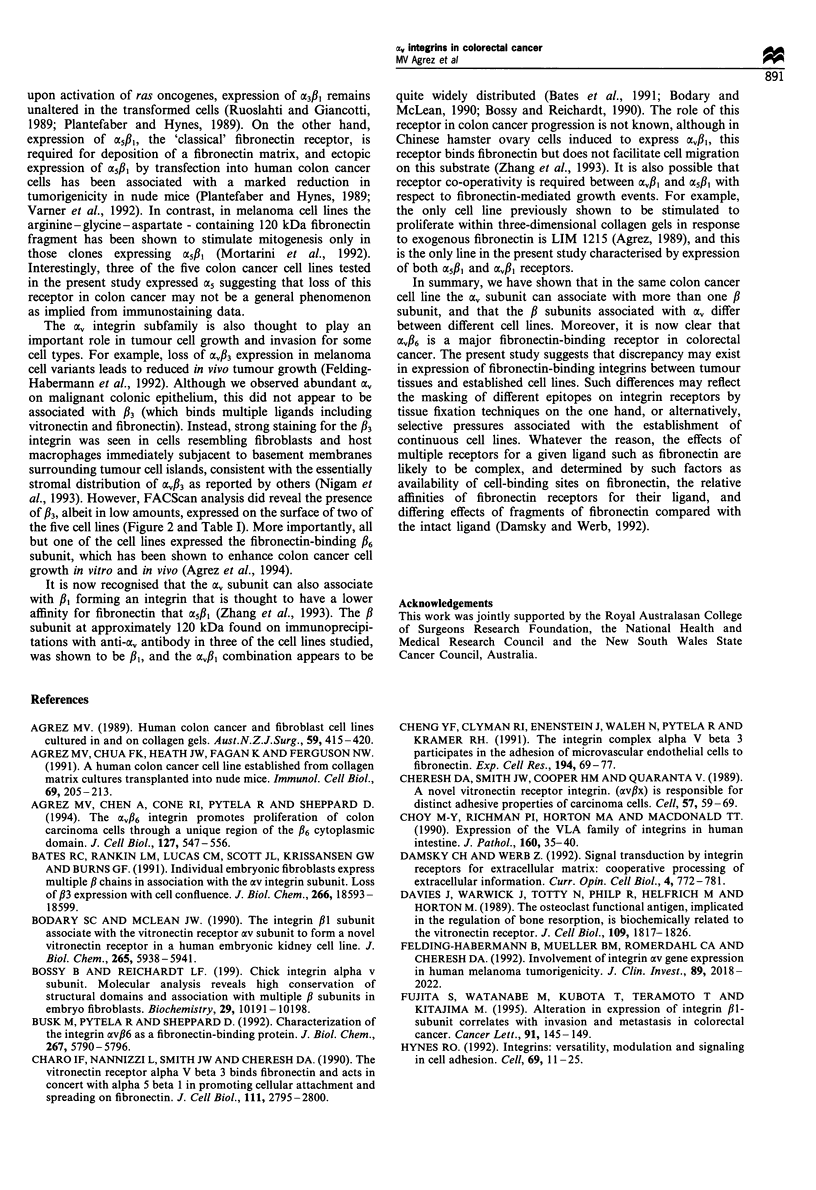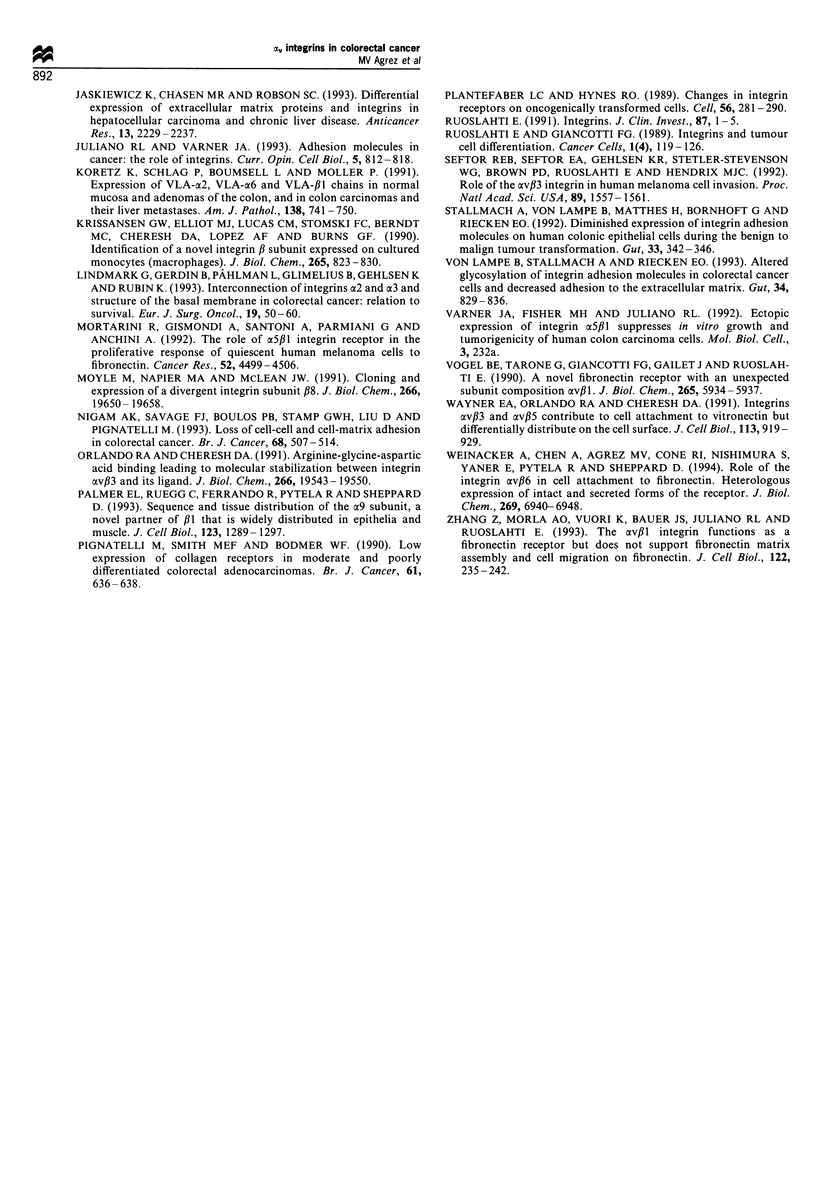# Multiplicity of fibronectin-binding alpha V integrin receptors in colorectal cancer.

**DOI:** 10.1038/bjc.1996.158

**Published:** 1996-04

**Authors:** M. V. Agrez, R. C. Bates, D. Mitchell, N. Wilson, N. Ferguson, P. Anseline, D. Sheppard

**Affiliations:** Discipline of Surgical Science, Cancer Research Unit, John Hunter Hospital, Newcastle, Australia.

## Abstract

**Images:**


British Journal of Cancer (1996) 73, 887-892
© 1996 Stockton Press All rights reserved 0007-0920/96 $12.00

### Multiplicity of fibronectin-binding $\alpha_v$ integrin receptors in colorectal cancer

MV Agrez[1], RC Bates[2], D Mitchell[1], N Wilson[3], N Ferguson[3], P Anseline[4] and D Sheppard[5]

[1]*Discipline of Surgical Science,* [2]*Cancer Research Unit,* [3] *Department of Pathology,* [4] *Department of Surgery, John Hunter Hospital, Newcastle, Australia;* [5]*Lung Biology Center, University of San Francisco, USA.*

**Summary** Current data from *in vitro* and *in vivo* animal models indicate that fibronectin-binding integrin receptors expressed by colon cancer cells may regulate tumour growth. While individual members of the $\beta_1$ subfamily of integrins have now been clearly identified in colorectal cancer, little information exists with respect to the $\alpha_v$ subfamily. In the present study we show that $\alpha_v$ can associate with multiple and different $\beta$ subunits capable of binding fibronectin in this tumour type. This is likely to have functional implications for growth and spread of colorectal cancer.

**Keywords:** colorectal cancer; fibronectin; integrins

The ability of tumour cells to invade surrounding tissues is a fundamental characteristic of malignant neoplasms. Extracellular matrices play a significant role in regulating the behaviour and differentiation of neoplastic cells during this process. These matrices include various collagens and non-collagenous glycoproteins such as laminin and fibronectin, which exert their effect through an array of cell membrane receptors, the best characterised of which is a family of transmembrane molecules termed integrins (Hynes, 1992).

The integrin receptors are heterodimeric complexes of an alpha ($\alpha$) and beta ($\beta$) subunit in non-covalent association. Excluding the leucocyte integrins, which are designated by the $\beta_2$ nomenclature, and which mediate cell-cell interactions, integrins can be divided into two major groups. The $\beta_1$ subfamily, also designated VLA (very late antigen), defines one group in which the $\beta_1$ chain combines with nine $\alpha$ chain members ($\alpha_{1-9}$) (Palmer *et al.*, 1993). Within this subfamily, integrins that bind fibronectin are $\alpha_5\beta_1$, $\alpha_4\beta_1$ and $\alpha_3\beta_1$ (Hynes, 1992). The $\alpha_4$ subunit is restricted mostly to lymphoid and myeloid cells and immunostaining of human colon cancers has not revealed the presence of $\alpha_4\beta_1$ (Stallmach *et al.*, 1992). However, other members of the $\beta_1$ subfamily of integrins ($\alpha_2\beta_1$, $\alpha_3\beta_1$, $\alpha_5\beta_1$ and $\alpha_6\beta_1$ are expressed in abundance in the normal colon, although individual $\beta_1$ integrins may be lost during tumour progression (Choy *et al.*, 1990; Koretz *et al.*, 1991; Stallmach *et al.*, 1992; Nigam *et al.*, 1993; Lindmark *et al.*, 1993). A systematic study of integrin expression in normal colon, adenomas and carcinomas within the same patients has shown progressive loss of expression of $\alpha_3\beta_1$ (which also recognises laminin and collagen) and $\alpha_5\beta_1$ (which recognises fibronectin only) in the transition from adenomas to carcinomas (Pignatelli *et al.*, 1990; Stallmach *et al.*, 1992). In one report 75% of colorectal carcinomas were found to be completely negative for expression of the major VLA fibronectin receptor, $\alpha_5\beta_1$ (Stallmach *et al.*, 1992).

The other subfamily of integrins, designated $\alpha_v$, is so named because the $\alpha_v$ subunit is capable of associating with multiple $\beta$ subunits, including $\beta_1$, $\beta_3$, $\beta_5$, $\beta_6$ and $\beta_8$ in various cell lines, but the primary functional role of this subfamily in cancer progression is still unclear (Cheresh *et al.*, 1989; Bodary and McLean, 1990; Vogel *et al.*, 1990; Orlando and Cheresh, 1991; Wayner *et al.*, 1991; Moyle *et al.*, 1991; Busk *et al.*, 1992). Of these receptors, $\alpha_v\beta_1$, $\alpha_v\beta_3$ and $\alpha_v\beta_6$ and perhaps $\alpha_v\beta_5$ are capable of binding fibronectin (Hynes, 1992; Zhang *et al.*, 1993; Charo *et al.*, 1990; Cheng *et al.*, 1991; Busk *et al.*, 1992; Weinacker *et al.*, 1994). Some clues as to the function of the $\alpha_v$ subfamily of receptors in tumorigenesis have come from melanoma studies in which the presence of the $\alpha_v\beta_3$ integrin (which binds multiple ligands, including fibronectin) has been shown to correlate with enhanced invasiveness of melanoma cells *in vitro* and increased melanoma growth *in vivo* (Seftor *et al.*, 1992). In contrast, the $\beta_3$ subunit has either not been identified at all in normal and malignant epithelium (Stallmach *et al.*, 1992) or found to be weakly expressed in a minority of tissue specimens (Nigam *et al.*, 1993). On the other hand weak expression of $\alpha_v$ has been reported for the majority of normal mucosal specimens and half of the tumours in one series of patients (Nigam *et al.*, 1993).

It has been suggested that expression of fibronectin receptors and the assembly of fibronectin matrix is closely associated with a tumorigenic phenotype (Ruoslahti, 1991). For example, in hepatocellular and breast carcinomas a correlation has been established between poorer differentiation of tumours and up-regulation of expression of some extracellular matrix molecules including fibronectin (Jaskiewicz *et al.*, 1993). These matrix changes have been observed at the interface between tumour and invaded tissues, suggesting a role for these matrix molecules in invasive and growth-stimulatory events. Indeed, we have previously reported that fibronectin can stimulate growth of colon cancer cells cultured within three-dimensional collagen matrices (Agrez, 1989). These findings are consistent with the recent proposal that the major fibronectin-binding integrin of the $\beta_1$ subfamily, $\alpha_5\beta_1$, functions as a negative growth regulator when not bound to its ligand, whereas when the receptor is occupied the negative signal is relieved and/or a positive growth signal is generated (Juliano and Varner, 1993). Moreover, recent data from our laboratory have shown that one member of the $\alpha_v$ subfamily that binds fibronectin, $\alpha_v\beta_6$, also enhances growth of colon cancer cells *in vitro* and *in vivo* (Agrez *et al.*, 1994). In the present study we present our characterisation of fibronectin-binding $\alpha_v$ integrins on colon cancer cells, and consider the functional implications of receptor redundancy in this tumour type.

### Methods and materials

### *Cell lines*

Three colon cancer cell lines, COLO 205, SW480 and WiDr, were obtained from the American Type Culture Collection (ATCC, Rockville, MD, USA). Another line, designated 020588, has previously been established in our laboratory (Agrez *et al.*, 1991) and another cancer cell line, LIM 1215, was a generous gift from Dr R Whithead, Ludwig Institute of Cancer Research, Melbourne, Australia. All lines were adapted to monolayer growth in tissue culture flasks using standard culture medium consisting of Dulbecco's modified

Correspondence: MV Agrez, Discipline of Surgical Science, Faculty of Medicine and Health Sciences, Level 4, David Maddison Building, Royal Newcastle Hospital, Newcastle, New South Wales 2300, Australia
Received 22 March 1995; revised 9 October 1995; accepted 27 October 1995

eagle's medium (DMEM) (Flow Laboratories, VA, USA) supplemented with glutamine, antibiotics (penicillin and streptomycin) and 10% fetal calf serum.

*Antibodies and peptides*

Monoclonal antibodies (MAbs) to the $\alpha_v$ subunit (13C2) and $\alpha_v\beta_3$ complex (23C6) (Davies *et al.*, 1989) were a kind gift from Dr Michael Horton of the Imperial Cancer Research Fund, London, and anti-$\beta_3$ subunit (SZ21) was a kind gift from Dr Michael Berndt of the Baker Institute, Melbourne, Australia. Antibody to the $\beta_1$ subunit (QE.2E5) was a kind gift from Dr Grahame Russ of the Queen Elizabeth Hospital, Adelaide, Australia. Monoclonal antibodies against $\beta_5$ (PIF6) and $\beta_6$ (E7P6) were prepared as previously described (Weinacker *et al.*, 1994). Antibody against $\alpha_3$ (P1B5) was obtained from Telios (San Diego, CA, USA) and antibody against $\alpha_5$ (SAM-1) was supplied by Immunotech, France. The polyclonal anti-$\beta_1$ antibody 3847, was a kind gift from Dr Ken Yamada (NIH). The polyclonal anti-human vitronectin receptor (anti-VNR) antibody was purchased from GIBCO BRL (Grand Island, NY, USA).

*Immunostaining of resected colon specimens*

*Immunofluorescence* Fresh tissue blocks were taken from non-necrotic areas of colon cancers and mounted in OCT compound (Miles), snap frozen in liquid nitrogen and cut in a Microm cryostat (Zeiss, West Germany). Frozen sections 5 $\mu$M thick were placed on glass slides coated with 0.1% poly-L-lysine (Sigma, St Louis, MO, USA) and fixed briefly (30 s) in chilled acetone. Sections were washed with phosphate-buffered saline (PBS) and incubated with optimally diluted primary antibody in a humid chamber for 30 min. After washing with PBS for 5 min, sections were then incubated in the dark with FITC-conjugated anti-mouse IgG (Dako, Denmark). Sections were mounted using glycerol/PBS 9:1 (v/v) pH 8.6 containing 2.5% DACO [1,4-diazobicyclo (2.2.2) octane; Aldrich Chemical Co., Milwaukee, WI, USA] to inhibit quenching of fluorescence. Negative controls using irrelevant antibody were run with each batch of slides. The slides were examined using a Nikon Optiphot microscope with fluorescence and UFX camera attachments.

*Immunoperoxidase* Sections were cut and fixed as for fluorescence staining. Before staining, sections were treated with 3% hydrogen peroxide to quench endogenous peroxidase activity, washed in PBS and incubated in non-immune goat serum (Dako, Denmark) for 20 min. Without washing, excess serum was removed and optimally diluted antibodies applied for 30 min. All incubations were carried out in a humid chamber at room temperature. After a brief wash in PBS, sections were sequentially incubated for 30 min in biotinylated anti-mouse IgG (1:200, Dako, Denmark) and preformed streptavidin–biotin complex (Dako K377). The presence of antigen was visualised by staining with DAB (5 mg 3,3 diaminobenzidine tetrahydrochloride; Sigma, in 10 ml of 0.95 M Tris-HCl buffer pH 7.6 with 10 $\mu$l of 30% hydrogen peroxide) for 5 min. Sections were counterstained with Harris haematoxylin, dehydrated and mounted with Ultramount (Fronine, Riverstone, Australia). Appropriate positive and negative controls were run in parallel with each batch of slides. The slides were examined on a Nikon Optiphot microscope.

*Cell staining and flow cytometric analysis* Cells from each of the five tumour cell lines were harvested from monolayer cultures using 20 mM EDTA, washed once with DMEM, and resuspended in normal goat serum at 4°C for 10 min to block non-specific binding of the secondary antibody (goat anti-mouse IgG). The cells were then incubated with the candidate antibodies for 20 min at 4°C and washed twice with PBS. The cell mixture was then stained with secondary antibody conjugated to phycoerythrin (Boehringer Mannheim) for 20 min at 4°C, washed twice with PBS and resuspended in PBS for analysis with FACScan (Becton Dickinson, Rutherford, NJ, USA).

**Figure 1** (**a**) Immunohistological staining of the $\alpha_v$ integrin chain in normal mucosa (left) and in a colon adenocarcinoma (right) using the 13C2 monoclonal antibody (magnification ×200). (**b**) Immunohistological staining of the $\beta_3$ subunit in a colon adenocarcinoma using the SZ21 monoclonal antibody (magnification ×400). (**c**) Immunohistological staining of the $\beta_6$ subunit in normal mucosa (left) and in a colon adenocarcinoma (right) using the E7P6 monoclonal antibody (magnification ×100).

*Immunoprecipitation* Cells were labelled at the cell surface by lactoperoxidase-catalysed iodination, essentially as previously described (Krissansen *et al.*, 1990). The cells were then washed three times to remove unbound label and solubilised in octylglucoside lysis buffer (100 mmol 1-0-*N*-octyl-$\beta$-D-glucopyranoside, 10 $\mu$M Tris, 150 $\mu$M sodium chloride, 2 $\mu$M phenylmethylsulphonyl fluoride (PMSF), 20

μM iodoacetamide and 50 μg $ml^{-1}$ soybean trypsin inhibitor) for 1 h on ice. The lysates were centrifuged at 10 000 g for 10 min, then precleared with RAM (rabbit anti-mouse immunoglobulin) coupled to Sepharose 4B beads for 2 h. Immunoprecipitations were carried out using antibodies directly coupled to Sepharose 4B and analysed by sodium dodecyl sulphate - polyacrylamide gel electrophoresis (SDS-PAGE) (7.5% gel) and autoradiography as described previously (Bates *et al.*, 1991).

*Immunoblotting* Cell lysates were immunoprecipitated essentially as described above, however, protein-A coupled-Sepharose 4B beads were used with the polyclonal anti-VNR and anti-$\beta_1$ antibodies. Immunoprecipitates were subjected to non-reducing 7.5% SDS-PAGE, then electrophoretically transferred to nitrocellulose membrane. The membrane was probed with the anti-$\beta_1$ MAb QE.2E5, and developed using an HRP-conjugated goat anti-mouse secondary (Bio-Rad, Hercules, CA, USA), followed by enhanced chemiluminescence (ECL) detection (DuPont NEN, Boston, MA, USA).

### Results

### *Immunostaining of fresh tissue specimens*

Initially, tissue sections were prepared from three primary colorectal malignancies and tested for the distribution of $\alpha_v$ and $\beta_3$ using the MAbs 13C2 and SZ21 respectively. Immunofluorescent preparations of all sections with antibody against $\alpha_v$ revealed abundant expression of the $\alpha_v$ subunit in tumour cells as well as in normal mucosa (Figure 1a). In contrast, the $\beta_3$ subunit was detected only in stromal cells within interstitial planes between tumour cell islands, and in none of the sections was $\beta_3$ expressed on tumour cells (Figure 1b).

A further 12 primary tumours were stained with MAb E7P6 against the $\beta_6$ integrin subunit (fibronectin and tenascin receptor). $\beta_6$ was identified in 6 of these 12 specimens. In each tumour, patchy expression was restricted to the epithelial component and in some cases, expression of $\beta_6$ was most prominent within malignant cells located at the advancing edges of tumour cell islands (Figure 1c). There was no correlation between the amount of $\beta_6$ staining in tumour specimens and either histological grade or clinico-pathological stage of the cancers.

### *FACScan analyses*

To determine whether established human colon cancer cell lines also displayed $\alpha_v$ but lacked surface expression of the $\beta_3$ integrin, we examined a panel of 5 tumour cell lines by means of FACScan analysis. The fluorescent profiles for two

**Figure 2** Fluorescence-activated cell analyses histograms showing expression of integrin subunits in two human colon cancer cell lines (COLO205 and SW480). $\alpha_v$ was detected with monoclonal antibody 13C2; $\beta_1$ with QE2E5; $\alpha_v\beta_3$ complex with 23C6; $\beta_5$ with PIF6; and $\beta_6$with E7P6.

**Figure 3** Fluorescence-activated cell analyses histograms showing expression of $\alpha_5$ and $\beta_6$ subunits in five human colon cancer cell lines (COLO205, SW480, WiDr, LIM1215 and 020588). $\alpha_5$ was detected with monoclonal antibody SAM-1, and $\beta_6$ with E7P6.

representative cell lines, COLO 205 and SW480 are shown in Figure 2, and it is clear that these cells express $\alpha_v$ in association with various $\beta$ subunits on the cell surface. COLO 205 expressed high levels of $\alpha_v$ integrin, as well as the $\beta_5$ subunit (primarily a vitronectin receptor) and $\beta_6$ subunit, but no $\beta_3$. In contrast, SW480 cells displayed $\alpha_v$ expression on the cell surface in association with $\beta_5$ but this line lacked expression of $\beta_6$. The SW480 cells also expressed the $\beta_3$ subunit as the $\alpha_v\beta_3$ complex – however, this expression was more than an order of magnitude lower than that of the $\beta_5$ subunit. All four remaining cell lines expressed $\beta_6$ as shown in Figure 3. The pattern of integrin expression shown for the COLO 205 cells, $\alpha_v$ in association with both $\beta_5$ and $\beta_6$ subunits in the absence of $\beta_3$, was the same for the cell lines LIM 1215 and 020588, while the remaining line WiDr expressed both $\beta_5$ and $\beta_6$ as well as low levels of the $\beta_3$ subunit (see Table I).

Although $\beta_1$ was expressed on the surface of all five cell lines (see Table I), expression of $\alpha_5$ was restricted to three lines, COLO 205, LIM 1215 and SW480 as shown in Figure 3. In contrast, all cell lines were strongly positive for $\alpha_3$ and on none of the lines was there detectable expression of the $\alpha_4$ subunit (data not shown).

*Immunoprecipitation analysis*

To confirm the association of $\alpha_v$ with different $\beta$ subunits and to determine whether this subunit also associated with $\beta_1$ on colon cancer cells, immunoprecipitations of lysates of cells surface-labelled with $^{125}$I were performed with the monoclonal antibody 13C2 against $\alpha_v$ subunits. The results confirmed the presence of the $\alpha_v$ integrin in association with multiple and varied $\beta$ subunits (Figure 4a) on all five cell lines tested. In three cell lines, designated 020588, LIM 1215 and WiDr, but not in SW480 and COLO 205 cells, a band of approximately 120 kDa co-precipitated with $\alpha_v$ (Figure 4a). To confirm the physical association of $\alpha_v$ with $\beta_1$ in these three tumour cell lines, cell lysates were immunoprecipitated with anti-$\alpha_v$ antibody and the transferred proteins immunoblotted with anti-$\beta_1$ antibody. A prominent band at approximately 120 kDa was identified on immunoblots (Figure 4b) that migrated to the same position as $\beta_1$ in control immunoblots of LIM1215 and SW480 cells that had been immunoprecipitated with anti-$\beta_1$ antibody (Figure 4c). The lower band in Figure 4c most likely represents the pre-$\beta_1$ chain previously identified in human colonic carcinomas (von Lampe *et al.*, 1993; Fujita *et al.*, 1995). In contrast, immunoprecipitation and immunoblotting of lysates from SW480 and COLO 205 cells did not show $\beta_1$ in association with $\alpha_v$ (Figure 4b). Taken together with the FACScan results, it was apparent that at least two $\alpha_v$ integrin receptors potentially capable of binding fibronectin ($\alpha_v\beta_1$, $\alpha_v\beta_3$, $\alpha_v\beta_5$ or $\alpha_v\beta_6$) were present on each of the colon cancer cell lines tested.

**Table I** FACScan analyses of expression of various integrin subunits on human colon cancer cell lines

| | *SW480* | *COLO 205* | *020588* | *WiDr* | *LIM1215* |
|---|---|---|---|---|---|
| $\alpha_v$ | + | + | + | + | + |
| $\beta_1$ | + | + | + | + | + |
| $\beta_3$ | + | − | − | + | − |
| $\beta_5$ | + | + | + | + | + |
| $\beta_6$ | − | + | + | + | + |

### Discussion

Tumour growth, invasion and metastasis is likely to be determined by the balance between available integrin receptors present on tumour cells and the nature of the surrounding matrix environment. For example, in the $\beta_1$ integrin subfamily, significant loss of tissue staining for two fibronectin-binding integrins, $\alpha_3$ and $\alpha_5$, has been reported in colorectal cancers (Pignatelli *et al.*, 1990; Stallmach *et al.*, 1992). The effect of $\alpha_3$ occupancy by fibronectin on tumour cell growth is not known. It has been suggested that $\alpha_3$ has a lower affinity for fibronectin than $\alpha_5$, and in contrast to the diminished expression of $\alpha_5$ by rodent fibroblasts consequent

**Figure 4** (**a**) Immunoprecipitation analysis of colon carcinoma cell lines. Cells were surface labelled with $^{125}$I, immunoprecipitated with the anti-$\alpha_v$ MAb 13C2, and analysed by SDS-PAGE and autoradiography. Precipitation of $\alpha_v$ complexes are shown for all five lines under non-reducing conditions. Varied expression and multiple $\beta$ subunits are seen complexed with $\alpha_v$. (**b**) Immunoblot analysis of $\alpha_v$-defined complexes from colon carcinoma cell lines. The same five cell lines were immunoprecipitated with an anti-$\alpha_v$ complex antibody, transferred to nitrocellulose and probed with the anti-$\beta_1$ MAb QE.2E5. The identity of the approximately 120 kDa band associating with $\alpha_v$ shown in (**a**) for lines 020588, LIM1215 and WiDr is thus confirmed as $\beta_1$. (**c**) $\beta_1$ immunoblot controls for $\alpha_v\beta_1$-expressing and -non-expressing cell lines (LIM1215 and SW480 respectively). The two lines were immunoprecipitated with polyclonal $\beta_1$ antibody and probed with QE.2E5, as in **b**.

upon activation of *ras* oncogenes, expression of $\alpha_3\beta_1$ remains unaltered in the transformed cells (Ruoslahti and Giancotti, 1989; Plantefaber and Hynes, 1989). On the other hand, expression of $\alpha_5\beta_1$, the 'classical' fibronectin receptor, is required for deposition of a fibronectin matrix, and ectopic expression of $\alpha_5\beta_1$ by transfection into human colon cancer cells has been associated with a marked reduction in tumorigenicity in nude mice (Plantefaber and Hynes, 1989; Varner *et al.*, 1992). In contrast, in melanoma cell lines the arginine–glycine–aspartate - containing 120 kDa fibronectin fragment has been shown to stimulate mitogenesis only in those clones expressing $\alpha_5\beta_1$ (Mortarini *et al.*, 1992). Interestingly, three of the five colon cancer cell lines tested in the present study expressed $\alpha_5$ suggesting that loss of this receptor in colon cancer may not be a general phenomenon as implied from immunostaining data.

The $\alpha_v$ integrin subfamily is also thought to play an important role in tumour cell growth and invasion for some cell types. For example, loss of $\alpha_v\beta_3$ expression in melanoma cell variants leads to reduced *in vivo* tumour growth (Felding-Habermann *et al.*, 1992). Although we observed abundant $\alpha_v$ on malignant colonic epithelium, this did not appear to be associated with $\beta_3$ (which binds multiple ligands including vitronectin and fibronectin). Instead, strong staining for the $\beta_3$ integrin was seen in cells resembling fibroblasts and host macrophages immediately subjacent to basement membranes surrounding tumour cell islands, consistent with the essentially stromal distribution of $\alpha_v\beta_3$ as reported by others (Nigam *et al.*, 1993). However, FACScan analysis did reveal the presence of $\beta_3$, albeit in low amounts, expressed on the surface of two of the five cell lines (Figure 2 and Table I). More importantly, all but one of the cell lines expressed the fibronectin-binding $\beta_6$ subunit, which has been shown to enhance colon cancer cell growth *in vitro* and *in vivo* (Agrez et al., 1994).

It is now recognised that the $\alpha_v$ subunit can also associate with $\beta_1$ forming an integrin that is thought to have a lower affinity for fibronectin that $\alpha_5\beta_1$ (Zhang *et al.*, 1993). The $\beta$ subunit at approximately 120 kDa found on immunoprecipitations with anti-$\alpha_v$ antibody in three of the cell lines studied, was shown to be $\beta_1$, and the $\alpha_v\beta_1$ combination appears to be quite widely distributed (Bates *et al.*, 1991; Bodary and McLean, 1990; Bossy and Reichardt, 1990). The role of this receptor in colon cancer progression is not known, although in Chinese hamster ovary cells induced to express $\alpha_v\beta_1$, this receptor binds fibronectin but does not facilitate cell migration on this substrate (Zhang *et al.*, 1993). It is also possible that receptor co-operativity is required between $\alpha_v\beta_1$ and $\alpha_5\beta_1$ with respect to fibronectin-mediated growth events. For example, the only cell line previously shown to be stimulated to proliferate within three-dimensional collagen gels in response to exogenous fibronectin is LIM 1215 (Agrez, 1989), and this is the only line in the present study characterised by expression of both $\alpha_5\beta_1$ and $\alpha_v\beta_1$ receptors.

In summary, we have shown that in the same colon cancer cell line the $\alpha_v$ subunit can associate with more than one $\beta$ subunit, and that the $\beta$ subunits associated with $\alpha_v$ differ between different cell lines. Moreover, it is now clear that $\alpha_v\beta_6$ is a major fibronectin-binding receptor in colorectal cancer. The present study suggests that discrepancy may exist in expression of fibronectin-binding integrins between tumour tissues and established cell lines. Such differences may reflect the masking of different epitopes on integrin receptors by tissue fixation techniques on the one hand, or alternatively, selective pressures associated with the establishment of continuous cell lines. Whatever the reason, the effects of multiple receptors for a given ligand such as fibronectin are likely to be complex, and determined by such factors as availability of cell-binding sites on fibronectin, the relative affinities of fibronectin receptors for their ligand, and differing effects of fragments of fibronectin compared with the intact ligand (Damsky and Werb, 1992).

**Acknowledgements**
This work was jointly supported by the Royal Australasan College of Surgeons Research Foundation, the National Health and Medical Research Council and the New South Wales State Cancer Council, Australia.

### References

AGREZ MV. (1989). Human colon cancer and fibroblast cell lines cultured in and on collagen gels. *Aust.N.Z.J.Surg.*, **59,** 415–420.

AGREZ MV, CHUA FK, HEATH JW, FAGAN K AND FERGUSON NW. (1991). A human colon cancer cell line established from collagen matrix cultures transplanted into nude mice. *Immunol. Cell Biol.*, **69,** 205–213.

AGREZ MV, CHEN A, CONE RI, PYTELA R AND SHEPPARD D. (1994). The $\alpha_v\beta_6$ integrin promotes proliferation of colon carcinoma cells through a unique region of the $\beta_6$ cytoplasmic domain. *J. Cell Biol.*, **127,** 547–556.

BATES RC, RANKIN LM, LUCAS CM, SCOTT JL, KRISSANSEN GW AND BURNS GF. (1991). Individual embryonic fibroblasts express multiple $\beta$ chains in association with the $\alpha$v integrin subunit. Loss of $\beta$3 expression with cell confluence. *J. Biol. Chem.*, **266,** 18593–18599.

BODARY SC AND MCLEAN JW. (1990). The integrin $\beta$1 subunit associate with the vitronectin receptor $\alpha$v subunit to form a novel vitronectin receptor in a human embryonic kidney cell line. *J. Biol. Chem.*, **265,** 5938–5941.

BOSSY B AND REICHARDT LF. (199). Chick integrin alpha v subunit. Molecular analysis reveals high conservation of structural domains and association with multiple $\beta$ subunits in embryo fibroblasts. *Biochemistry*, **29,** 10191–10198.

BUSK M, PYTELA R AND SHEPPARD D. (1992). Characterization of the integrin $\alpha$v$\beta$6 as a fibronectin-binding protein. *J. Biol. Chem.*, **267,** 5790–5796.

CHARO IF, NANNIZZI L, SMITH JW AND CHERESH DA. (1990). The vitronectin receptor alpha V beta 3 binds fibronectin and acts in concert with alpha 5 beta 1 in promoting cellular attachment and spreading on fibronectin. *J. Cell Biol.*, **111,** 2795–2800.

CHENG YF, CLYMAN RI, ENENSTEIN J, WALEH N, PYTELA R AND KRAMER RH. (1991). The integrin complex alpha V beta 3 participates in the adhesion of microvascular endothelial cells to fibronectin. *Exp. Cell Res.*, **194,** 69–77.

CHERESH DA, SMITH JW, COOPER HM AND QUARANTA V. (1989). A novel vitronectin receptor integrin. ($\alpha$v$\beta$x) is responsible for distinct adhesive properties of carcinoma cells. *Cell*, **57,** 59–69.

CHOY M-Y, RICHMAN PI, HORTON MA AND MACDONALD TT. (1990). Expression of the VLA family of integrins in human intestine. *J. Pathol.*, **160,** 35–40.

DAMSKY CH AND WERB Z. (1992). Signal transduction by integrin receptors for extracellular matrix: cooperative processing of extracellular information. *Curr. Opin. Cell Biol.*, **4,** 772–781.

DAVIES J, WARWICK J, TOTTY N, PHILP R, HELFRICH M AND HORTON M. (1989). The osteoclast functional antigen, implicated in the regulation of bone resorption, is biochemically related to the vitronectin receptor. *J. Cell Biol.*, **109,** 1817–1826.

FELDING-HABERMANN B, MUELLER BM, ROMERDAHL CA AND CHERESH DA. (1992). Involvement of integrin $\alpha$v gene expression in human melanoma tumorigenicity. *J. Clin. Invest.*, **89,** 2018–2022.

FUJITA S, WATANABE M, KUBOTA T, TERAMOTO T AND KITAJIMA M. (1995). Alteration in expression of integrin $\beta$1-subunit correlates with invasion and metastasis in colorectal cancer. *Cancer Lett.*, **91,** 145–149.

HYNES RO. (1992). Integrins: versatility, modulation and signaling in cell adhesion. *Cell*, **69,** 11–25.

JASKIEWICZ K, CHASEN MR AND ROBSON SC. (1993). Differential expression of extracellular matrix proteins and integrins in hepatocellular carcinoma and chronic liver disease. *Anticancer Res.*, **13**, 2229–2237.

JULIANO RL AND VARNER JA. (1993). Adhesion molecules in cancer: the role of integrins. *Curr. Opin. Cell Biol.*, **5**, 812–818.

KORETZ K, SCHLAG P, BOUMSELL L AND MOLLER P. (1991). Expression of VLA-$\alpha$2, VLA-$\alpha$6 and VLA-$\beta$1 chains in normal mucosa and adenomas of the colon, and in colon carcinomas and their liver metastases. *Am. J. Pathol.*, **138**, 741–750.

KRISSANSEN GW, ELLIOT MJ, LUCAS CM, STOMSKI FC, BERNDT MC, CHERESH DA, LOPEZ AF AND BURNS GF. (1990). Identification of a novel integrin $\beta$ subunit expressed on cultured monocytes (macrophages). *J. Biol. Chem.*, **265**, 823–830.

LINDMARK G, GERDIN B, PÅHLMAN L, GLIMELIUS B, GEHLSEN K AND RUBIN K. (1993). Interconnection of integrins $\alpha$2 and $\alpha$3 and structure of the basal membrane in colorectal cancer: relation to survival. *Eur. J. Surg. Oncol.*, **19**, 50–60.

MORTARINI R, GISMONDI A, SANTONI A, PARMIANI G AND ANCHINI A. (1992). The role of $\alpha5\beta1$ integrin receptor in the proliferative response of quiescent human melanoma cells to fibronectin. *Cancer Res.*, **52**, 4499–4506.

MOYLE M, NAPIER MA AND MCLEAN JW. (1991). Cloning and expression of a divergent integrin subunit $\beta$8. *J. Biol. Chem.*, **266**, 19650–19658.

NIGAM AK, SAVAGE FJ, BOULOS PB, STAMP GWH, LIU D AND PIGNATELLI M. (1993). Loss of cell-cell and cell-matrix adhesion in colorectal cancer. *Br. J. Cancer*, **68**, 507–514.

ORLANDO RA AND CHERESH DA. (1991). Arginine-glycine-aspartic acid binding leading to molecular stabilization between integrin $\alpha v\beta3$ and its ligand. *J. Biol. Chem.*, **266**, 19543–19550.

PALMER EL, RUEGG C, FERRANDO R, PYTELA R AND SHEPPARD D. (1993). Sequence and tissue distribution of the $\alpha$9 subunit, a novel partner of $\beta$1 that is widely distributed in epithelia and muscle. *J. Cell Biol.*, **123**, 1289–1297.

PIGNATELLI M, SMITH MEF AND BODMER WF. (1990). Low expression of collagen receptors in moderate and poorly differentiated colorectal adenocarcinomas. *Br. J. Cancer*, **61**, 636–638.

PLANTEFABER LC AND HYNES RO. (1989). Changes in integrin receptors on oncogenically transformed cells. *Cell*, **56**, 281–290.

RUOSLAHTI E. (1991). Integrins. *J. Clin. Invest.*, **87**, 1–5.

RUOSLAHTI E AND GIANCOTTI FG. (1989). Integrins and tumour cell differentiation. *Cancer Cells*, **1(4)**, 119–126.

SEFTOR REB, SEFTOR EA, GEHLSEN KR, STETLER-STEVENSON WG, BROWN PD, RUOSLAHTI E AND HENDRIX MJC. (1992). Role of the $\alpha v\beta3$ integrin in human melanoma cell invasion. *Proc. Natl Acad. Sci. USA*, **89**, 1557–1561.

STALLMACH A, VON LAMPE B, MATTHES H, BORNHOFT G AND RIECKEN EO. (1992). Diminished expression of integrin adhesion molecules on human colonic epithelial cells during the benign to malign tumour transformation. *Gut*, **33**, 342–346.

VON LAMPE B, STALLMACH A AND RIECKEN EO. (1993). Altered glycosylation of integrin adhesion molecules in colorectal cancer cells and decreased adhesion to the extracellular matrix. *Gut*, **34**, 829–836.

VARNER JA, FISHER MH AND JULIANO RL. (1992). Ectopic expression of integrin $\alpha5\beta1$ suppresses *in vitro* growth and tumorigenicity of human colon carcinoma cells. *Mol. Biol. Cell.*, **3**, 232a.

VOGEL BE, TARONE G, GIANCOTTI FG, GAILET J AND RUOSLAHTI E. (1990). A novel fibronectin receptor with an unexpected subunit composition $\alpha v\beta1$. *J. Biol. Chem.*, **265**, 5934–5937.

WAYNER EA, ORLANDO RA AND CHERESH DA. (1991). Integrins $\alpha v\beta3$ and $\alpha v\beta5$ contribute to cell attachment to vitronectin but differentially distribute on the cell surface. *J. Cell Biol.*, **113**, 919–929.

WEINACKER A, CHEN A, AGREZ MV, CONE RI, NISHIMURA S, YANER E, PYTELA R AND SHEPPARD D. (1994). Role of the integrin $\alpha v\beta6$ in cell attachment to fibronectin. Heterologous expression of intact and secreted forms of the receptor. *J. Biol. Chem.*, **269**, 6940–6948.

ZHANG Z, MORLA AO, VUORI K, BAUER JS, JULIANO RL AND RUOSLAHTI E. (1993). The $\alpha v\beta1$ integrin functions as a fibronectin receptor but does not support fibronectin matrix assembly and cell migration on fibronectin. *J. Cell Biol.*, **122**, 235–242.